# Anti-Inflammatory Effect of *Dendrobium officinale* Extract on High-Fat Diet-Induced Obesity in Rats: Involvement of Gut Microbiota, Liver Transcriptomics, and NF-κB/IκB Pathway

**DOI:** 10.3390/antiox14040432

**Published:** 2025-04-03

**Authors:** Runze Zhou, Yixue Wang, Shiyun Chen, Fanjia Cheng, Yuhang Yi, Chenghao Lv, Si Qin

**Affiliations:** 1 College of Food Science and Technology, Hunan Agricultural University, Changsha 410128, China; zhourunze@stu.hunau.edu.cn (R.Z.); wangyixue@stu.hunau.edu.cn (Y.W.); shiyunchen0531@stu.hunau.edu.cn (S.C.); chengfanjia@stu.hunau.edu.cn (F.C.); 2 College of Bioscience and Biotechnology, Hunan Agricultural University, Changsha 410128, China; yiyuhang@stu.hunau.edu.cn; 3 Hunan Provincial Key Laboratory of Liver Visceral Manifestation in Traditional Chinese Medicine, Institute of Integrative Medicine, Department of Integrated Traditional Chinese and Western Medicine, Xiangya Hospital, Central South University, Changsha 410008, China

**Keywords:** *Dendrobium officinale*, oxidative stress, inflammation, gut microbiota, NF-κB/IκB pathway

## Abstract

The growing prevalence of obesity is being increasingly acknowledged as a major public health issue. This mainly stems from the excessive intake of dietary fats. *Dendrobium officinale* (DO), recognized as an herb with dual roles of food and medicine, is renowned for its diverse health-promoting effects. Nevertheless, the specifics of its antiobesity and anti-inflammatory properties and the underlying mechanisms are still obscure. The present study shows that treatment with *Dendrobium officinale* extract (DOE) alleviates obesity, liver steatosis, inflammation, and oxidative stress in rats that are obese due to a high-fat diet (HFD). Firstly, with respect to HFD obese rats, higher doses of DOE significantly reduced TG, TC, LDL-C, blood glucose, and liver AST and ALT, along with lipid droplets. Meanwhile, DOE supplementation significantly reduced oxidative stress induced by ROS and MDA and increased the levels of GSH-Px and SOD in liver tissues. Furthermore, integrated analysis of transcriptomic and microbiomic data revealed that DOE modulated inflammatory responses through the NF-κB/IκB pathway. This regulatory mechanism was evidenced by corresponding changes in the protein expression levels of both NF-κB and IκB. Additionally, DOE was found to modulate gut microbiota composition in obese rats, specifically reducing the relative abundance of *Bilophila* while increasing beneficial bacterial populations, particularly the genera *Akkermansia* and *Roseburia*. These findings suggest that DOE may help retain the homeostasis of the gut microbiota and improve metabolic health by regulating inflammation in the liver and intestine, thereby providing protection against obesity and related metabolic syndromes. Our study demonstrates that DOE, as a natural botanical extract, can effectively facilitate the prevention or treatment of metabolic syndrome through precision dietary interventions.

## 1. Introduction

During the last few decades, obesity has come to the fore as an acutely pressing global health challenge [1], contributing to numerous complications, including diabetes and other metabolic disorders [2]. Dyslipidemia stands as a crucial clinical characteristic of obesity. This condition involves profound disruptions in lipid metabolism. Specifically, it is characterized by a rise in triglyceride (TG) levels, an elevation of low-density lipoprotein cholesterol (LDL-C), and a reduction in high-density lipoprotein cholesterol (HDL-C) [3]. These alterations in lipid profiles not only serve as important diagnostic markers for obesity-related metabolic disorders but also contribute significantly to the heightened risk of cardiovascular diseases often associated with obesity. In addition to common metabolic disturbances, obesity and oxidative stress establish a bidirectional relationship where reactive oxygen species (ROS) drive systemic chronic inflammation, while chronic inflammation reciprocally impairs antioxidant defense systems [4]. Oxidative stress, a physiological state arising from the imbalance between ROS production and the antioxidant defense system, plays a pivotal role in exacerbating obesity-related complications. Cells rely on an intricate antioxidant system for maintaining oxidative equilibrium. This system encompasses enzymatic antioxidants like superoxide dismutases (SODs), catalase (CAT), and glutathione peroxidases (GPXs) [5]. However, in obesity, the persistent elevation of oxidative stress not only worsens lipid metabolism disorders but also perpetuates chronic inflammation, creating a vicious cycle. The intricate bidirectional interplay between oxidative stress and obesity acts as a potent catalyst in expediting the onset and progression of metabolic diseases. This is primarily due to the fact that each element within this relationship serves to exacerbate the pernicious impacts of the other, creating a self-perpetuating and escalating cycle of metabolic dysregulation. Moreover, recent studies emphasize the involvement of gut microbiota in this process [6], as dysbiosis can trigger systemic inflammation and further contribute to oxidative stress, thereby amplifying metabolic dysfunction.

The evolution and worsening of long-term health issues similar to obesity have a substantial connection with the arrangement and physiological performance of the microorganisms within the intestinal microbiome. A prolonged diet high in fat and sugar can cause an imbalance in the gut microbiota of obese individuals, which subsequently hampers intestinal defense mechanisms. Disrupted microbiota and their metabolic products pass through the intestinal barrier, resulting in insulin resistance, energy metabolism imbalance, white adipose browning process impairment, and elevated levels of inflammatory factors, which in turn lead to chronic diseases such as diabetes, hypertension, and cardiovascular disease [7,8]. The nuclear factor κB (NF-κB) is of great importance and acts as a key player as a transcription factor, overseeing the expression of proteins related to immune system responses [9]. In the process of canonical NF-κB signaling, the degradation of IκBs occurs via the ubiquitination–proteasome pathway, leading to the release of NF-κB dimers that were previously sequestered. As a result, these dimers move to the nucleus. This action prompts the genes encoding inflammatory cytokines and chemokines to be expressed. In this way, it sets off successive inflammatory reactions in the intestines and liver [10].

High throughput RNA sequencing (RNA-seq) technology can reveal the molecular mechanisms in specific biological processes and the occurrence processes of diseases, and has been widely applied in fields such as basic research, clinical diagnosis, and drug research and development. Recent studies have demonstrated that high-fat diet-fed rats not only exhibit upregulation of relevant inflammatory indicators but also mediate inflammatory responses through the NF-κB signaling pathway [11,12]. Moreover, *Dendrobium officinale* can also improve liver inflammation through the NF-κB pathway. Yang et al. [13] demonstrated that the extract of *Dendrobium officinale* could successfully inhibit the phosphorylation of NF-κB in a rat model of liver injury and alleviate ethanol-induced acute liver injury through the TLR4/NF-κB signaling pathway. Zhang et al. [14] demonstrated that transcription factors in the STAT/nuclear factor κB (NF-κB)/IRF families, in combination with miR-148a/375/9a, act as key regulators of inflammation and apoptosis pathways in type 2 diabetes mellitus (T2DM) rats treated with DOE. These studies indicate that DOE has a direct impact on the liver at the transcriptome level.

Recent studies have demonstrated that various plant extracts can mitigate obesity and its associated lipid metabolism disorders by having an impact on the abundance and composition of the gut microbiome [15]. *Dendrobium officinale*, which is extensively utilized both as a traditional Chinese medicine (TCM) and a food with medicine–food homology, is rich in a variety of compounds. These include polysaccharides, phenanthrenes, and bibenzyls [16], exhibiting diverse pharmacological effects, including reducing lipid levels, modulating the gut microbiota, safeguarding the liver, suppressing inflammation, lowering blood sugar, and maintaining intestinal health [17]. Additionally, research has shown that *D. officinale* polysaccharides (DOP) have the ability to influence gut bacteria to create short-chain fatty acids in the large intestines of mice [18]. Nevertheless, the available data on its anti-obesity effect and metabolic advantages are scarce, while the fundamental mechanisms remain even more obscure. In this study, our objective is to clarify the protective mechanisms of *Dendrobium officinale* extract (DOE) against obesity and related metabolic syndrome. In addition, our aim is to explore whether the gut microbiota plays an etiological role in promoting the beneficial effects of DOE on the metabolism of the host organism. Additionally, we plan to investigate its mechanism of action through hepatic transcriptomics, explore the specific role of DOE in the liver while verifying these findings using western blot technology, and aim to provide scientific evidence for its application in treating inflammation.

## 2. Materials and Methods

### 2.1. Extraction of Dendrobium Officinale

Fresh stems of *Dendrobium officinale* were collected from a local planting cooperative in Xinning County (Shaoyang, China). Their species status as *Dendrobium officinale* was confirmed through taxonomic identification by the research team led by Prof. Luo Yibo from the Institute of Botany, Chinese Academy of Sciences. The stem segments of the *Dendrobium officinale* raw materials were kept and put in an oven at 40 °C for 24 h. Then, a grinder was used to crush them into fine powder. After being screened through a 40-mesh sieve, the powder was stored in a −20 °C refrigerator. A quantity of 50 g of the dry powdered material was extracted in 60% ethanol with a solid-to-liquid ratio of 1:8 (*w*/*v*, at 60 °C for 60 min). After rotary evaporation, it was placed in an −80 °C refrigerator overnight, and then vacuum freeze-drying was conducted for 12 h to obtain the finished product.

### 2.2. HPLC-MS Analysis of DOE

Weigh 50 mg of *Dendrobium officinale* extract sample powder using an electronic balance (MS105DΜ, Mettler-Toledo International Inc., Zurich, Switzerland), and add 1200 μL of −20 °C pre-cooled 70% methanolic aqueous internal standard extract. Vortex once every 30 min for 30 s, for a total of 6 times. After centrifugation (rotation speed 12,000 rpm, 3 min), aspirate the supernatant, filter the sample through a microporous membrane (0.22 μm pore size), and store it in the injection vial for UPLC-MS analysis. One aliquot is analyzed under positive ion conditions and is eluted from a T3 column (Waters, Milford, MA, USA, ACQUITY Premier HSS T3 Column 1.8 µm, 2.1 mm × 100 mm) using 0.1% formic acid in water as solvent A and 0.1% formic acid in acetonitrile as solvent B in the following gradient: 5 to 20% in 2 min, increased to 60% in the following 3 min, increased to 99% in 1 min and held for 1.5 min, then return to 5% mobile phase B within 0.1 min, held for 2.4 min. The analytical conditions are as follows: column temperature, 40 °C; flow rate, 0.4 mL/min; injection volume, 4 μL. Another aliquot is analyzed under negative ion conditions and has the same elution gradient as the positive mode (Figure S1).

### 2.3. Animals and Experiment Design

A sum of 25 male Sprague Dawley (SD) rats, each weighing 180–220 g, was purchased from Hunan Slyke Jingda Laboratory Animal Ltd. (Changsha, China). Then, these rats were housed in a controlled environment at the Hunan Center for Drug Safety Evaluation and Research, where the room temperature was maintained at 22 ± 2 °C, and the light–dark cycle was set to 12 h. Subsequently, the rats were assigned randomly into five groups (n = 5 per group), namely the control group; the HFD group; the simvastatin (SIM) group (statins, particularly simvastatin, are selected as the positive control group due to their lipid-lowering effects through reducing LDL-C) [19]; the low-dose *Dendrobium officinale* (LDOE) group; and the high-dose *Dendrobium officinale* (HDOE) group. Control was supplied with a basal chow diet (SCXK<Jing>2019-0003, Beijing Ke Ao Xie Li Feed Co. Ltd., Beijing, China), and other groups were supplied with an HFD (TP23300, containing 19.4% crude protein, 60.0% crude fat, and 20.6% carbohydrate, TROPHIC Animal Feed High-Tech Co., Ltd., Nantong, China) for 12 weeks.

Among them, one group was administered simvastatin (SIM) at a dosage of 0.002 g/kg of body weight (based on the previous laboratory research on the positive drugs selected for establishing the hyperlipidemia model group, we chose a simvastatin dosage of 0.002 g/kg) [20]. Based on the previous basic laboratory research, the following administration groups were set up. Another group received a low-dose (LDOE) of Dendrobium officinale extract (DOE) at 0.54 g/kg of body weight, and the last group was given a high-dose (HDOE) of DOE at 1.08 g/kg of body weight. The oral gavage volume for each rat was 0.1 mL/10 g, and the treatment lasted for 12 weeks.

Throughout the experiment, all rats were provided unrestricted access to clean water and food. Weekly, the food consumption and body weights of the rats were closely monitored and accurately documented. After 12 weeks of dietary intervention, the rats were fasted for 12 h. Subsequently, their body weights, body lengths, and waist circumference were recorded. Then, they were administered chloral hydrate to induce anesthesia. Once the rats were anesthetized, blood and faeces were collected, after which the rats were euthanized. The small intestine tissue had been partially dissected and rinsed. The proximal colon part had been frozen in liquid nitrogen for subsequent experiments. The calculation formula of the Lee’s index [21] is shown in Equation (1):

(1) $$\mathrm{The}\;\mathrm{calculation}\;\mathrm{formula}\;\mathrm{of}\;\mathrm{the}\;\mathrm{Lee}’s\;\mathrm{index}=\sqrt[{3}]{\left\{body\;weight\left(g\right)\times 1000/body\;length\left(cm\right)\right\}}$$

### 2.4. Serum and Liver Biochemical Analysis

Samples of blood were taken from the orbital vein of rats and quickly transferred to 2-mL centrifuge tubes for preservation. The samples were positioned at room temperature and allowed to remain stationary for a duration of 30 min. Subsequently, they were centrifuged at 3500× *g* for 10 min. After that, the supernatant, namely the serum, was carefully collected and stored at −80 °C. These serum samples were utilized to measure the levels of total cholesterol (TC), triglycerides (TG), high-density lipoprotein cholesterol (HDL-C), and low-density lipoprotein cholesterol (LDL-C). Blood glucose levels were determined using blood glucose test strips. Furthermore, 100 mg of liver tissue was homogenized and then thoroughly mixed with 1 mL of ice-cold extract (The composition of the cold extract employed in each kit is presented in Table S1). The mixture was centrifuged at 8000× *g* for 10 min. The resulting supernatant was collected to analyze the enzyme levels of alanine transaminase (ALT), aspartate transaminase (AST), catalase (CAT), glutathione peroxidase (GSH-Px), superoxide dismutase (SOD), and the content of reactive oxygen species (ROS) in the liver. (The kits for measuring TC: TC-1-W, TG: TG-1-W, HDL-C: HDL-C-1-G, LDL-C: LDL-C-1-G, ALT: GPT-1-Y, AST: GOT-1-Y, CAT: CAT-1-W, GSH-Px: GPX-1-Y, SOD: SOD-1-W, and ROS: ROS-1-Y were all purchased from Suzhou Comin Biotechnology Co., Ltd., Suzhou, China.) All physiological and biochemical detection operations were executed with strict adherence to the instructions provided by commercial kits.

### 2.5. Histological Analysis and Morphometry

A portion of liver tissue was carefully dissected from each rat, and the tissue was embedded in optimal cutting temperature (OCT) compound (OCT: C0171A, a water-soluble mixture primarily composed of polyethylene glycol and polyvinyl alcohol, purchased from Beyotime Biotechnology Co., Ltd., Shanghai, China). The embedded tissue was then slowly immersed in liquid nitrogen. Using a cryostat maintained at −20 °C, continuous sections (10 μm thick) were cut. These sections were stained with hematoxylin-eosin (H&E) for histological examination to analyze hepatocyte morphology and the number of inflammatory cells. Finally, pathological imaging was performed using the DFC420C pathological imaging system (Leica, Wetzlar, Germany).

### 2.6. Liver RNA-Sequencing Analysis

After rats were sacrificed and portion of liver tissue was extracted, a part of the tissue was stored in Trizol (Invitrogen, Waltham, CA, USA). Afterward, total RNA was extracted from liver tissue using Trizol, adhering to the manufacturer’s protocol, and its quality was checked with the Agilent 2100. A total of 5 ng purified RNA was used to construct a library with Illumina TruSeq RNA Sample Preparation Kit v2 (Illumina, San Diego, CA, USA), and the library was sequenced on Illumina HiSeqTM2500 (Shanghai South Gene Technology Co., Ltd. Shanghai, China). Clean data were retrieved from the raw data by eliminating reads that contained adapters and low-quality reads through the FASTX-Toolkit (http://anaconda.org/bioconda/fastx_toolkit, accessed on 1 April 2025). The clean reads were aligned to the reference gene by making use of Bowtie software (https://bowtie-bio.sourceforge.net/index.shtml, accessed on 1 April 2025)and to the reference genome by employing the HISAT software (https://www.ccb.jhu.edu/software/hisat/index.shtml, accessed on 1 April 2025). In terms of gene expression analysis, the matched reads were computed and normalized to reads per kilobase of transcript per million by means of the RESM software (https://anaconda.org/bioconda/rsem, accessed on 1 April 2025). Differentially expressed genes (DEGs) were selected using the MA-plot-based approach with a random sampling model (MARS) and a false discovery rate threshold of less than 0.001, where the difference was regarded as significant. Gene ontology analysis was carried out by using the Gene Ontology Database (http://www.geneontology.org/, accessed on 1 April 2025), and Kyoto Encyclopedia of Genes and Genomes (KEGG); pathway annotation and enrichment analyses were founded on the KEGG pathway database (http://www.genome.jp/kegg/, accessed on 1 April 2025).

### 2.7. Western Blot Anlysis

The liver and intestinal tissue proteins were prepared according to the instructions of the Total Protein Extraction Kit (Solarbio Science and Technology Co., Ltd., Beijing, China). The samples were then centrifuged at 4 °C. Subsequently, the supernatant was collected, and the BCA Protein Assay Kit (Biosharp, Hefei, China) was employed to determine the protein concentration. Next, the protein extracts were carefully loaded onto a 10% SDS-PAGE gel. They underwent electrophoresis for proper separation and were then precisely transferred onto PVDF membranes (Amersham Pharmacia Biotech, Amersham, UK). Afterward, the membranes were incubated with skim milk at room temperature for an hour. After that, they were incubated overnight with the primary antibody at 4 °C. The primary antibodies against IκBα(4814S), Phospho-IκBα (P-IκBα, Ser32, 2859S), NF-κB(8242S), Phospho-NFκB (P-NFκB, Ser536,3033S) and β-actin (4967S), which were sourced from Cell Signaling Technology (CST, Waltham, MA, USA), were utilized at a dilution of 1:1000. Then, they were incubated for an additional 1 h with secondary antibody. The secondary antibody, anti-rabbit (Sigma-Aldrich, St. Louis, MI, USA), was used at a dilution of 1:30,000. The images were photographed and developed by Image Quant LAS 4000 mini (General Electric Company, Morrison, CO, USA). Software like ImageJ (https://imagej.net/ij/, accessed on 1 April 2025) was used to quantify the protein bands.

### 2.8. 16S rDNA Sequence Analysis

The collected feces samples had their DNA extracted using the E.Z.N.A.^®^ Stool DNA Kit (D4015, Omega Bio-tek, Inc., Norcross, GA, USA). Nuclease-free water was utilized as a control in the blank group to eliminate potential interference from nucleic acids during the experiment. The DNA underwent a thorough elution process with 50 µL of elution buffer, resulting in its complete release from the matrix. Afterward, the eluted DNA was carefully placed in storage at −80 °C to prevent any potential degradation. Primers 515F (5′-GTGYCAGCMGCCGCGGTAA-3′) and 805R (5′-GGACTACHVGGGTWTCTAAT-3′) can be utilized to amplify the prokaryotic small subunit (16S rRNA genes V3–V4 region). The primers’ 5′ ends were labeled with unique barcodes for each sample, and sequencing was conducted on the universal primers. For the PCR amplification procedure, a total reaction volume of 25 µL was carefully chosen to ensure optimal reaction conditions and reliable experimental results. To attain the specified final volume, this reaction mixture incorporated 25 ng of template DNA, PCR pre-mixture, 2.5 µL of each primer, and PCR-grade water. To amplify the prokaryotic 16S fragment, the PCR protocol was meticulously designed. It commenced with an initial denaturation phase at 98 °C for 30 s. Subsequently, 32 amplification cycles were executed, each consisting of a 10 s denaturation step at 98 °C, a 30 s annealing step at 54 °C, and a 45 s extension step at 72 °C. After the cycling, a final extension was performed at 72 °C for 10 min to ensure complete synthesis of the PCR products.

To validate the PCR products, electrophoresis was carried out using an agarose gel with a concentration of at least 2%. This high-concentration gel facilitated clear separation and visualization of the amplified fragments. During the entire DNA extraction procedure, ultrapure water was employed instead of the sample solution. This served as a negative control, a crucial measure to preclude false-positive results in the subsequent PCR analysis. By doing so, the reliability and accuracy of the experimental findings were effectively enhanced. Subsequently, the PCR products were purified using AMPure XP beads (Beckman Coulter Genomics, Danvers, MA, USA) and then accurately quantified with a Qubit 3.0 fluorometer. For sequencing preparation, amplification pools were established. To gain a comprehensive understanding of the amplified libraries, the size was meticulously analyzed using an Agilent 2100 Bioanalyzer (Agilent Technologies, Colorado Springs, CO, USA), while the quantity was precisely determined with an Illumina library quantification kit.

### 2.9. Statistical Analysis

StringTie 2.2.0 software was used to perform FPKM quantification of gene or transcript library data, and the R package edgeR (https://bioconductor.org/packages/release/bioc/html/edgeR.html, accessed on 1 April 2025)was used to analyze the differentially expressed genes between samples, with differences >2 times or <0.5 times (*p* < 0.05). DAVID (https://david.ncifcrf.gov/, accessed on 31 May 2023) was used to perform GO and KEGG enrichment analysis of genes.

Each independent experiment and data were analyzed and processed by SPSS26.0 software. Finally, GraphPad Prism 9 was used for graphics and text. All results are expressed as the mean of the number of experiments ± standard error of the mean (SEM). Significance analysis was performed using one-way ANOVA with multiple comparisons, and a statistical probability of *p* < 0.05 was considered statistically significant (* *p* < 0.05, ** *p* < 0.01, *** *p* < 0.001, **** *p* < 0.0001).

## 3. Results

### 3.1. Metabolite Analysis of DOE

A total of 21 classes of compounds were identified. Among them, amino acids and derivatives accounted for 22.66%, benzene and substituted derivatives accounted for 12.63%, organic acids accounted for 11.14%, alcohol and amines accounted for 5.32%, glycerophospholipids (GP) accounted for 4.84%, alkaloids accounted for 4.36%, heterocyclic compounds accounted for 4.24%, terpenoids accounted for 3.59%, lipids accounted for 2.82%, flavonoids accounted for 2.73%, phenolic acids accounted for 2.46%, nucleotides and derivatives accounted for 1.75%, glycerides (GL) accounted for 1.6%, fatty acyls (FA) accounted for 1.54%, lignans and coumarins accounted for 1.29%, steroids accounted for 0.72%, sphingolipids (SL) accounted for 0.48%, quinones accounted for 0.19%, tryptamines, cholines, and pigments accounted for 0.13%, tannins accounted for 0.1%, and others accounted for 15.4%. Among them, flavonoids, terpenoids, alkaloids (Table S2) and other components in *Dendrobium officinale* have been reported in many literatures, and they have functions such as anti-inflammation and antioxidation [22,23].

### 3.2. The Effects of DOE on the Physiological Indexes of High-Fat Diet Rats

**Figure 1:**
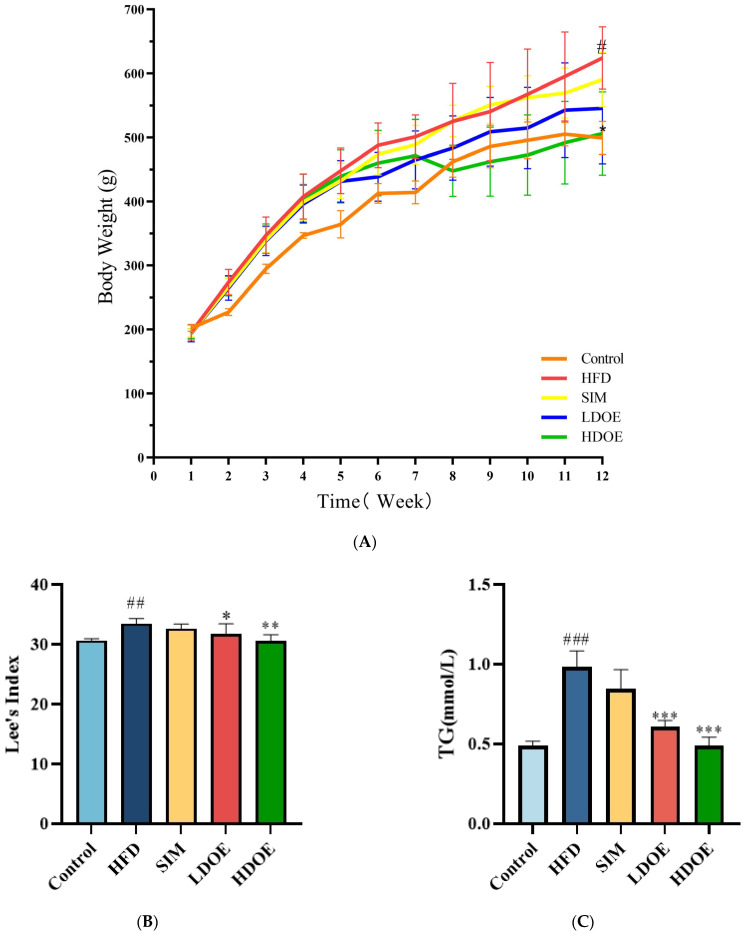

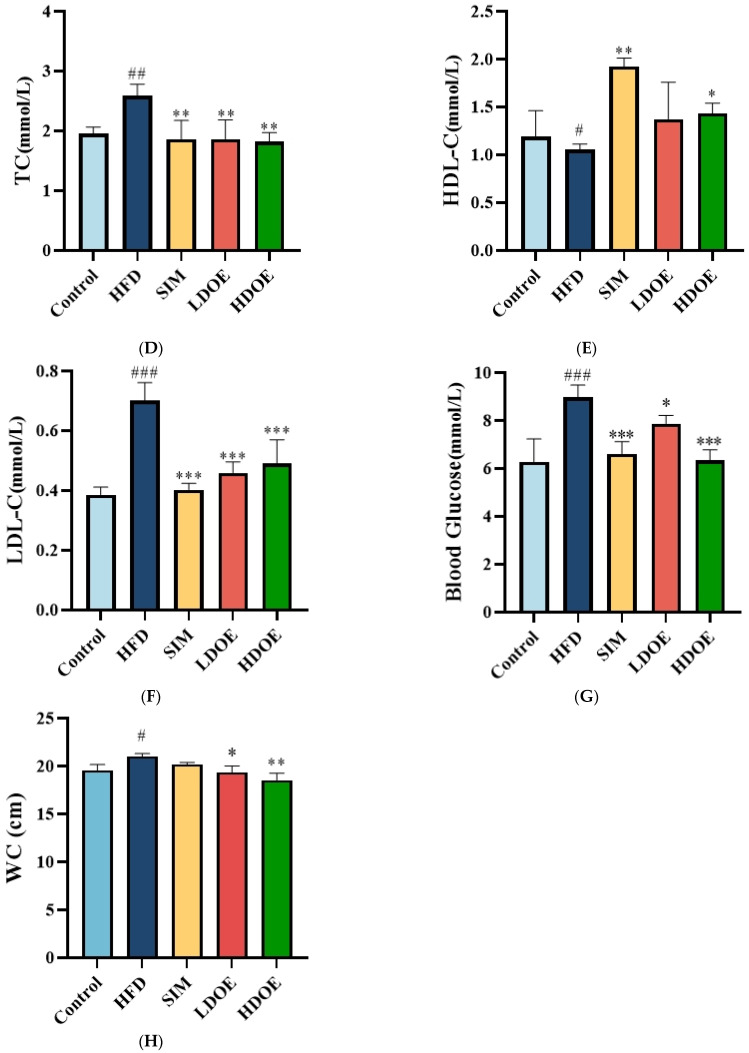
The effect of DOE on the physiological indexes of HFD rats: (**A**) Body weight; (**B**) Lee’s Index; (**C**) TG; (**D**) TC; (**E**) HDL-C; (**F**) LDL-C; (**G**) Blood glucose; (**H**) Waist circumference (WC). Control vs. HFD, *p* < 0.05, #; *p* < 0.01, ##; *p* < 0.005, ###; SIM, LDOE, HDOE vs. HFD, *p* < 0.05, *; *p* < 0.01, **; *p* < 0.005, ***.

At the end of the 12-week experiment (Figure 1A), the weight range of the control is 499.3 ± 25.9, and the weight range of the HFD is 610.8 ± 67.7. The weight of the SIM group is 590.5 ± 42.6, and the weights of the LDOE group and the HDOE group are 545.6 ± 86.9 and 506.3 ± 65.4, respectively. Compared with the control group, the HFD group showed a significant increase in body weight (*p* < 0.05); compared with the HFD group, the HDOE group significantly reduced body weight in rats (*p* < 0.05). To specifically evaluate whether the model establishment in the HFD group is successful or not, the Lee’s index needs to be used for determination. The Lee’s index is an important indicator for evaluating the degree of obesity in animals. By comparing each group based on the Lee’s index (Figure 1B), it is found that the Lee’s index of the HFD group is significantly higher than that of the control group (*p* < 0.01), which indicates that the rat obesity model has been successfully established. Compared with the HFD group, the Lee’s indices of the LDOE group and the HDOE group are significantly decreased (*p* < 0.05 and *p* < 0.01), suggesting that DOE has an intervening effect on rat obesity.

Compared to the control group, the HFD group significantly elevated TG (Figure 1C), TC (Figure 1D), LDL-C (Figure 1F), waist circumference, and blood glucose levels, while the HDOE group substantially diminished TG, TC, LDL-C, and blood glucose levels (*p* < 0.01 and *p* < 0.001) and increased HDL-C (Figure 1E) levels. In terms of blood glucose, the HDOE group (*p* < 0.001) had a better down-regulating effect compared to the LDOE group (*p* < 0.01). Collectively, these results revealed that DOE could alleviate the weight gain induced by a HFD and adjust the physiological indexes.

### 3.3. Effects of DOE on Morphology of Liver Tissue and Hepatic Enzyme in High-Fat Diet Rats

**Figure 2:**
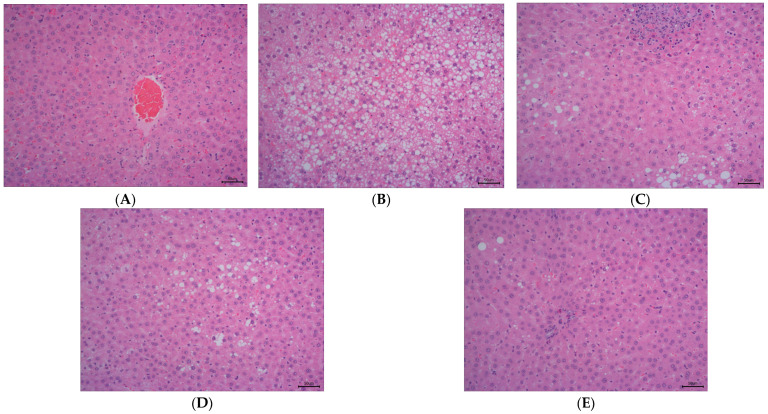

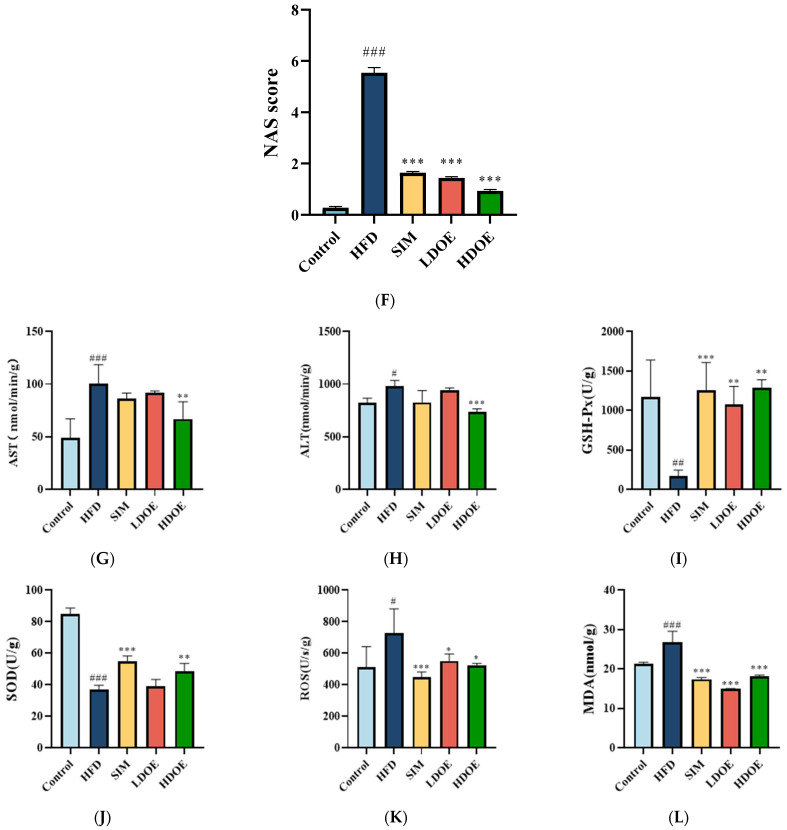
The effect of DOE on liver morphology in HFD rats. The liver tissue was stained with H&E and observed at 200×: (**A**) Control; (**B**) HFD; (**C**) SIM; (**D**) LDOE; (**E**) HDOE; (**F**) NAS score; (**G**) ALT; (**H**) AST; (**I**) GSH-Px; (**J**) SOD, (**K**) ROS; (**L**) MDA. HFD vs. Control, *p* < 0.05, #; *p* < 0.01, ##; *p* < 0.001, ###; SIM, LDOE, HDOE vs. HFD, *p* < 0.05, *; *p* < 0.01, **; *p* < 0.001, ***.

As illustrated in Figure 2, the staining method of H&E staining outcomes of liver tissue suggests that the hepatocytes in the control group (Figure 2A) have intact and distinct morphological structures, with no obvious vacuolar degeneration, and display typical cell morphology. Conversely, the livers of rats in the HFD group (Figure 2B) showed a notable rise in the quantity of lipid droplets and severe vacuolar degeneration.

The NAFLD activity score (NAS) is widely used to grade parenchymal alterations and is determined according to the criteria of Kleiner [24]. It is calculated based on the numerical score attributed to steatosis (0–3), hepatocyte ballooning (1–2), and lobular inflammation (0–3), as histologically investigated on fixed liver slices from the analyzed rats. As shown in Figure 2F, compared with the HFD, the NAS of rats treated with DOE was significantly decreased (*p* < 0.001). As demonstrated in Figure 2D–F, DOE could efficiently relieve the hepatic pathological alterations caused by HFD and safeguard the integrity of liver cell structure and function. When compared with the control group, the HFD group displayed substantial elevations in the levels of AST, ALT, ROS, and MDA (*p* < 0.05 and *p* < 0.001), along with marked declines in the levels of GSH-Px and SOD (*p* < 0.01 and *p* < 0.001). In contrast, when pitted against the HFD group, rats in the HDOE group demonstrated significant decreases in the levels of AST, ALT, ROS, and MDA (*p* < 0.05, *p* < 0.01, and *p* < 0.001), and notable increases in the levels of GSH-Px and SOD (*p* < 0.01). Collectively, these findings strongly imply that DOE can alleviate liver damage induced by an HFD.

### 3.4. Effect of DOE on Liver Transcriptomics in HFD-Fed Rats

**Figure 3:**
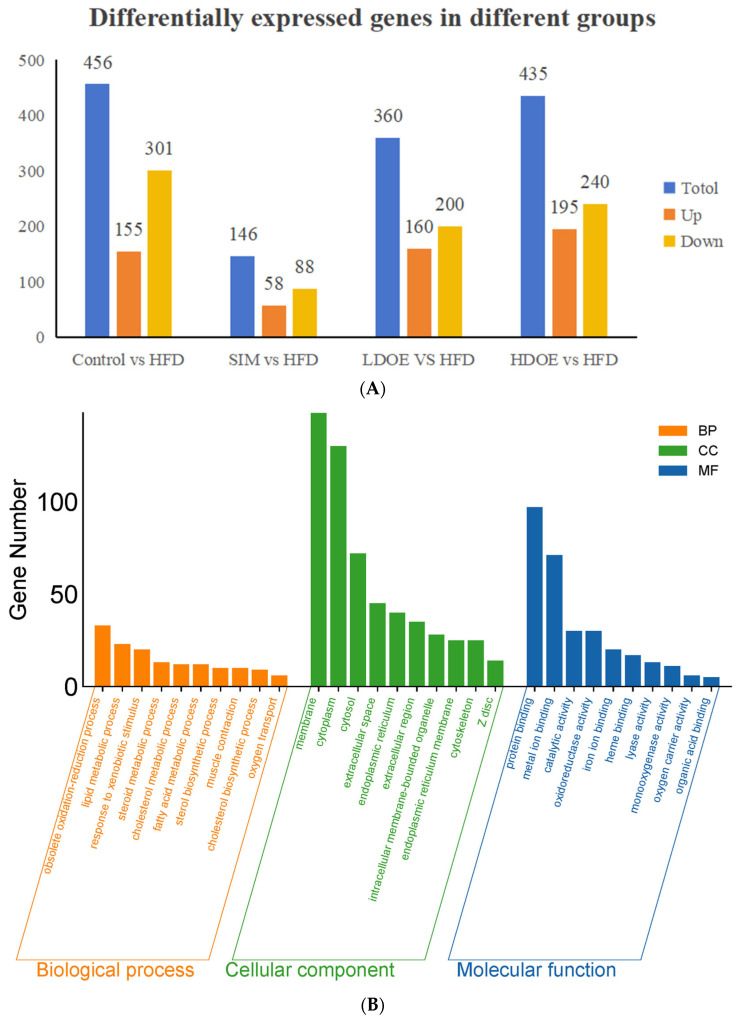

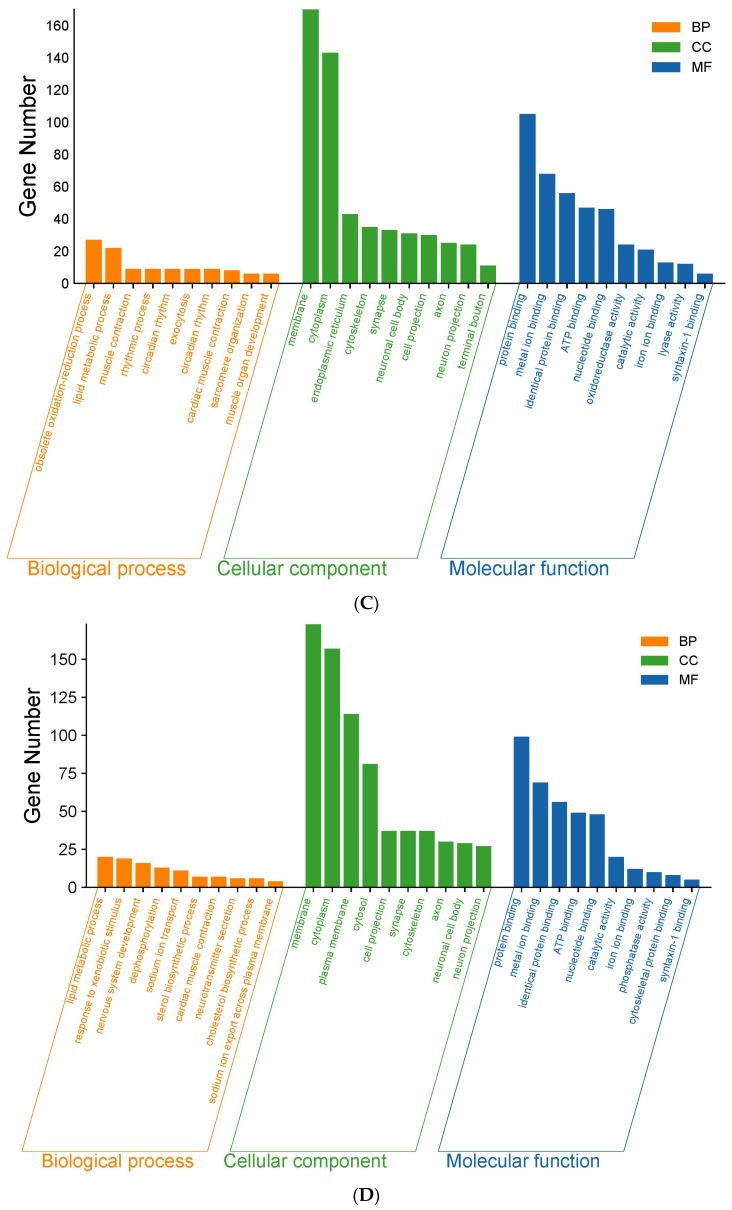

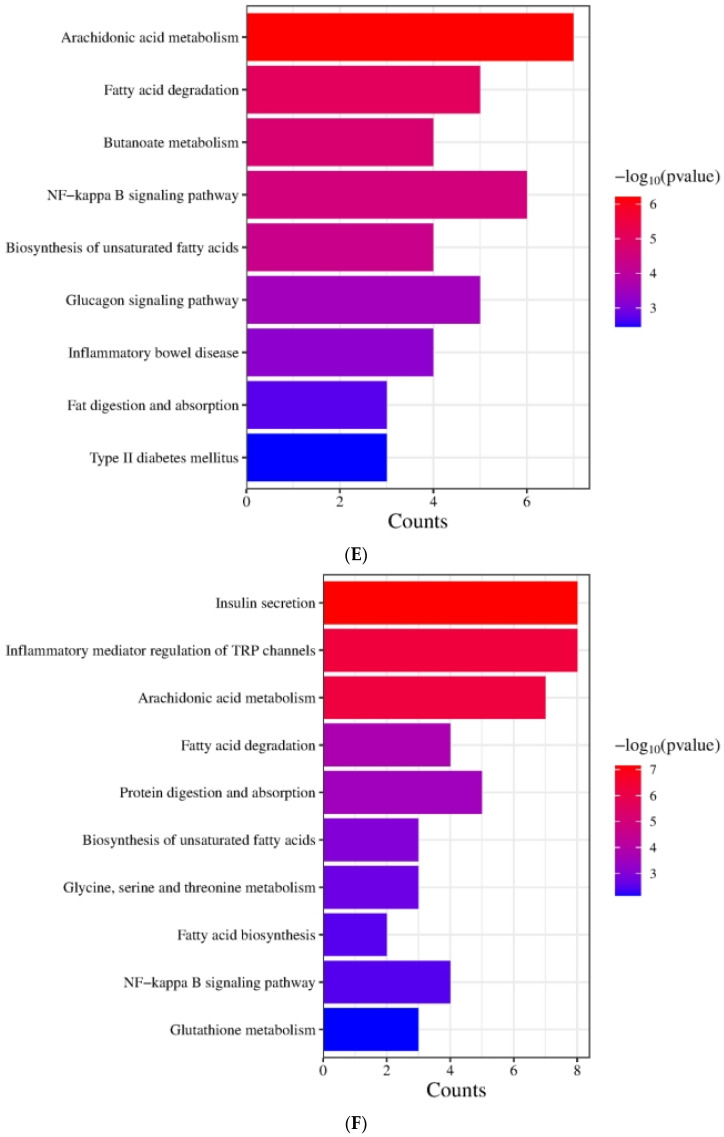

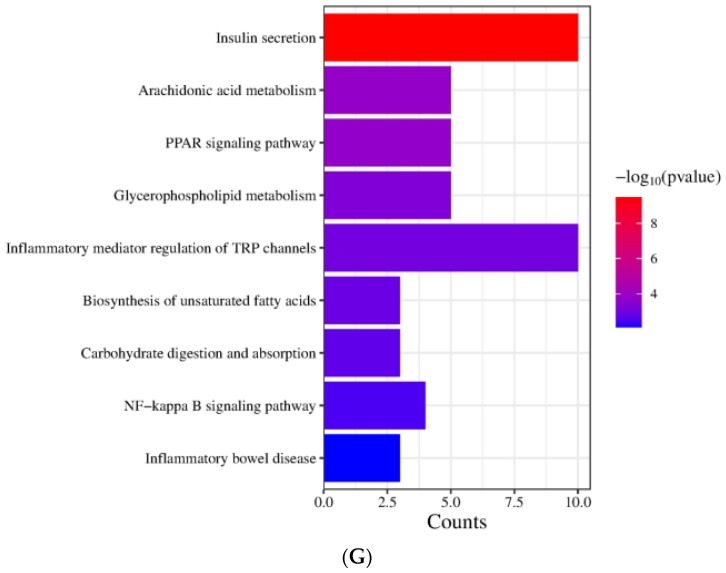
The effect of DOE on gene expressions in the liver of SD rats: (**A**) Genes showing differential expression across different groups. (**B**) Go enrichment analysis on the basis of the liver transcriptome. HFD vs. Control. (**C**) Go enrichment analysis on the basis of the liver transcriptome. LDOE vs. HFD. (**D**) Go enrichment analysis on the basis of the liver transcriptome. HDOE vs. HFD. (**E**) KEGG enrichment analysis based on liver transcriptome. HFD vs. Control (**F**) Enrichment analysis of KEGG Grounded in liver transcriptome. LDOE vs. HFD. (**G**) Enrichment analysis of KEGG grounded in liver transcriptome. HDOE vs. HFD.

As shown in Figure 3A, the results indicated that there were varying numbers of expressed genes across groups. Between the control and the HFD, 456 genes were differentially expressed, with 155 being upregulated and 301 downregulated. When comparing the SIM and the HFD, 146 genes showed differential expression, consisting of 58 upregulated and 88 downregulated genes. For the LDOE and the HFD comparison, 360 genes were differentially expressed, including 160 upregulated and 200 downregulated ones. In the case of the HDOE and the HFD, 435 genes were differentially expressed, with 195 being upregulated and 240 downregulated.

As shown in Figure 3B, control was compared with the HFD. In biological process (BP), the top 10 most significant entries were obsolete oxidation–reduction process, lipid metabolic process, response to xenobiotic stimulus, steroid metabolic process, cholesterol metabolic process, fatty acid metabolic process, sterol biosynthetic process, muscle contraction, cholesterol biosynthetic process, and oxygen transport. In cell component (CC), the top 10 most significant entries were membrane, cytoplasm, cytosol, extracellular space, endoplasmic reticulum, extracellular region, intracellular membrance-bounded organelle, endoplasmic reticulum membrane, cytoskeleton, and Zdisc. In molecular function (MF), the top 10 most significant entries were protein binding, metal ion binding catalytic activity, oxidoreductase activity, iron ion binding, heme binding, lyase activity, monooxygenase activity oxygen carrier activity, and organic acid binding.

As shown in Figure 3C, LDOE was compared with the HFD. In BP, the top 10 most significant entries were obsolete oxidation–reduction process, lipid metabolic process, muscle contraction, rhythmic process, circadian rhythm, exocytosis, circadian rhythm, cardiac muscle contraction, sarcomere organization, muscle organ development. In CC, the top 10 most significant entries were membrane, cytoplasm, endoplasmic reticulum, cytoskeleton, synapse, neuronal cell body, cell projection, axon, neuron projection, terminal bouton. In MF, the top 10 most significant entries were protein binding, metal ion binding, identical protein binding, ATP binding, nucleotide binding, oxidoreductase activity, catalytic activity, iron ion binding, lyase activity, syntaxin-1 binding.

As shown in Figure 3D, HDOE was compared with the HFD. In BP, the top 10 most significant entries were obsolete lipid metabolic process, response to xenobiotic stimulus, nervous system development, dephosphorylation, sodium ion transport, sterol biosynthetic process, cardiac muscle contraction, neurotransmitter secretion, cholesterol biosynthetic process, sodium ion export across plasma membrane. In CC, the top 10 most significant entries were membrane, cytoplasm, plasma membrane, cytosol, cell projection, synapse, cytoskeleton, axon, neuronal cell body, neuron projection. In MF, the top 10 most significant entries were protein binding, metal ion binding, identical protein binding, ATP binding, nucleotide binding, catalytic activity, iron ion binding, phosphatase activity, cytoskeletal protein binding, syntaxin-1 binding.

Figure 3E demonstrates that the KEGG biological pathways significantly enriched in the HFD were arachidonic acid metabolism, fatty acid degradation, butanoate metabolism, NF-kappa B signaling pathway, biosynthesis of unsaturated fatty acids, glucagon signaling pathway, inflammatory bowel disease, fat digestion and absorption, and Type Ⅱ diabetes mellitus. As shown in Figure 3F the KEGG biological pathways significantly enriched in the LDOE were as follows: insulin secretion, inflammatory mediator regulation of TRP channels, arachidonic acid metabolism, fatty acid degradation, protein digestion and absorption, biosynthesis of unsaturated fatty acids, glycine, serine and threonine metabolism, fatty acid biosynthesis, NF-kappa B signaling pathway, glutathione metabolism. As depicted in Figure 3G, the KEGG biological pathways with significant enrichment in the HDOE were as follows: insulin secretion, arachidonic acid metabolism, PPAR signaling pathway, glycerophospholipid metabolism, inflammatory mediator regulation of TRP channels, biosynthesis of unsaturated fatty acids, carbohydrate digestion and absorption, NF-kappa B signaling pathway, and inflammatory bowel disease.

Relying on the findings from GO and KEGG analyses, it was found that DOE could notably influence functions and signaling pathways associated with oxidative stress, inflammation, and lipid metabolism. By means of treatment and intervention during the development of obesity, DOE exerts an impact on the functions of relevant molecules and the corresponding signaling pathways. Intriguingly, the NF-κB signaling pathway, a crucial inflammatory signaling pathway, was significantly enriched in the HFD, LDOE, and HDOE groups.

### 3.5. Effects of DOE on NF-κB/IκBα Signaling Pathway in High-Fat Diet Rats

**Figure 4:**
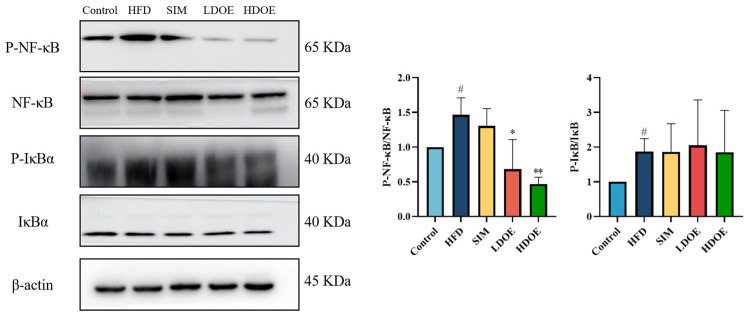
The effect of DOE on liver NF-κB/IκBα and their phosphorylated forms in the livers of HFD rats. HFD vs. Control, *p* < 0.05, #; SIM, LDOE, HDOE vs. HFD, *p* < 0.05, *; *p* < 0.01, **.

Chronic diseases such as obesity can lead to increased levels of inflammatory factors. Simultaneously, NF-κB serves as a key transcription factor that can influence the expression of proteins related to immune response. Analysis using western blot demonstrated (Figure 4) that the expression levels of NF-κB and IκBα proteins in the livers of rats on a HFD were notably increased (*p* < 0.05). In the HDOE group, the expression level of the NF-κB and IκBα protein had a downward trend, but IκBα not significantly. The above findings demonstrate that DOE can ameliorate HFD-induced liver injury and alleviate hepatic inflammation through the NF-κB signaling pathways.

### 3.6. Effect of DOE on the Gut Microbial Diversity of HFD Rats

With the aim of gaining a comprehensive understanding of the gut microbiota diversity in rats on an HFD, we employed the power of 16S rDNA gene sequencing techniques. Based on the abundance of characteristic values, we calculated the similarity and overlap of characteristic value compositions for each group and visualized the analysis through Venn diagrams. The results showed that 182 common components were present in the five groups, with the control group’s sequences still richer than the others. Compared to the HFD group, the differences in OTUs for the control group, SIM group, LDOE group, and HDOE group were 68.23%, 84.86%, 92.29%, and 77.36%, respectively, indicating different gut microbiota abundance among the groups.

**Figure 5:**
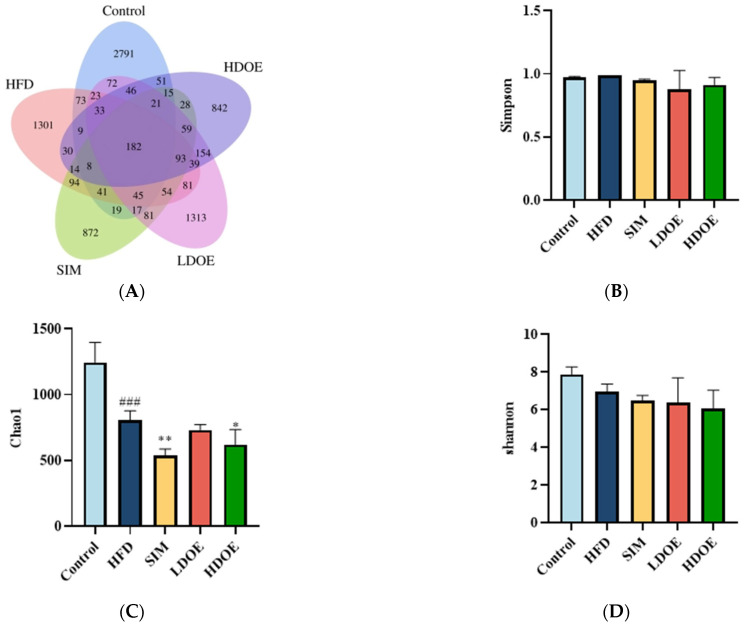
The effect of DOE on gut microbial diversity of HFD rats: (**A**) Venn diagram of different group based on OUT. (**B**) Simpson index. (**C**) Chao 1 index. (**D**) Shannon index. HFD vs. Control, *p* < 0.001, ###; SIM, LDOE, HDOE vs. HFD, *p* < 0.05, *; *p* < 0.01 **.

The variations in the alpha diversity of the gut microbiota community are displayed in Figure 5B–D). The Simpson index reflects the species richness in the gut microbiota. The Shannon index acts as an indicator to illustrate the evenness of a community. In contrast, the Chao 1 index is applied to measure the richness of the microbiota in the community. Compared to the control group, the Chao 1 index diminished in the other four groups (*p* < 0.05, *p* < 0.01, and *p* < 0.001). The control group exhibited a higher Shannon Index compared to other groups, though this difference was not statistically significant (Figure 5D). The results indicate that DOE treatment induces changes in the species diversity within HFD rats, specifically ameliorating the HFD-induced reduction in microbial species richness.

### 3.7. Effect of DOE on the Gut Microflora at the Phylum and Genus Level

**Figure 6:**
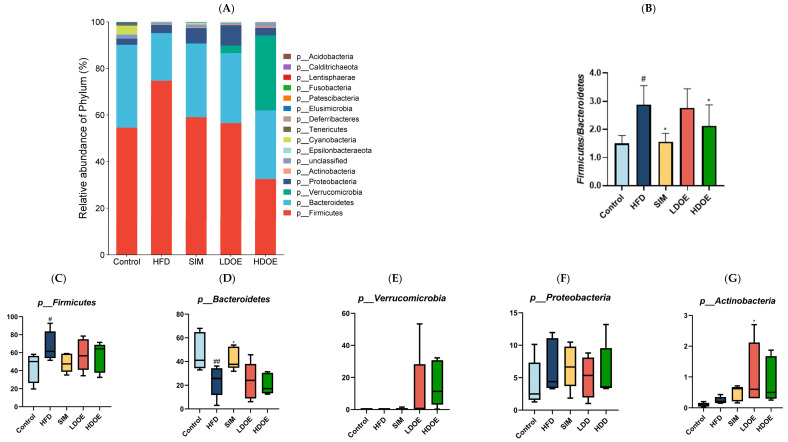

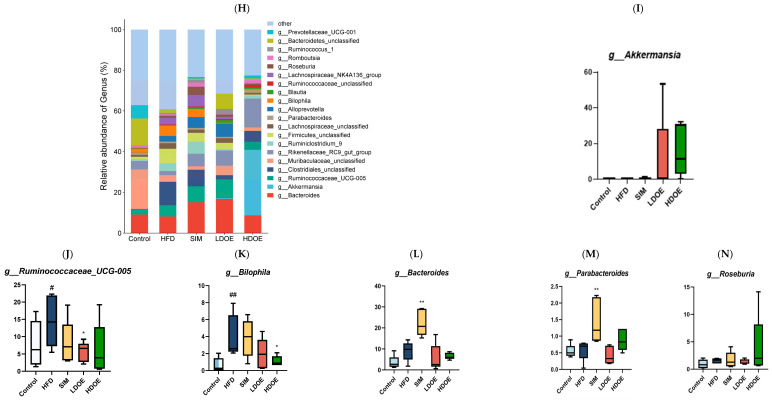

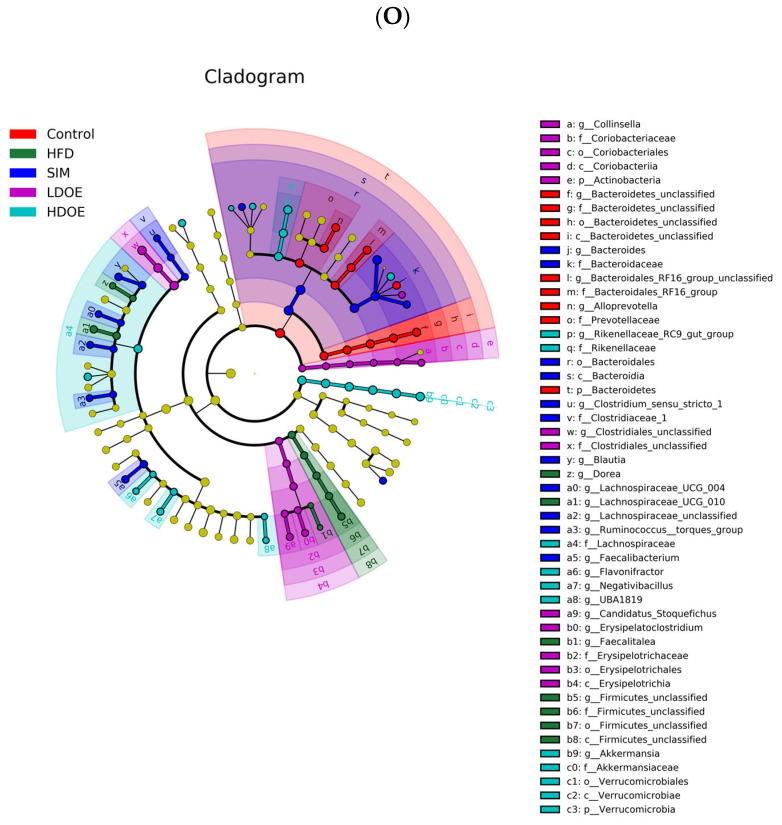

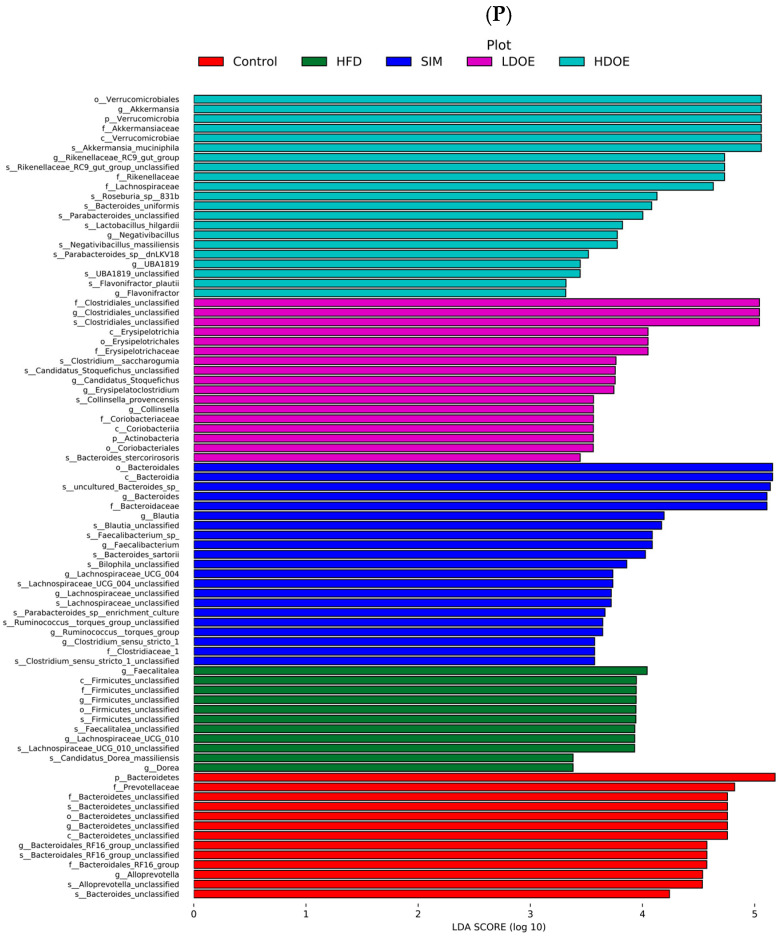

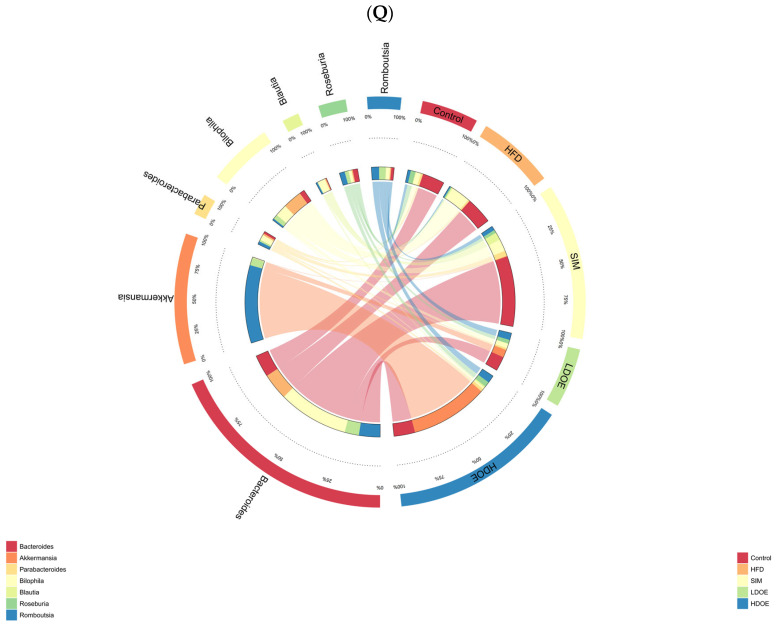
The effect of DOE on the phylum and genus level of intestinal microbiota in HFD rats: (**A**) Phylum level. (**B**) Abundance of *Firmicutes/Bacteroidetes.* (**C**) Box-plot of *Firmicutes* at the phylum level. (**D**) Box-plot of *Bacteroidetes* at the phylum level. (**E**) Box-plot of *Verrucomicrobia* at the level. (**F**) Box-plot of *Proteobacteria* at the phylum level. (**G**) Box-plot of *Deferribacteres* at the phylum level. (**H**) Genus level. (**I**) Box-plot of *Akkermansia* at the genus level. (**J**) Box-plot of *Ruminococcaceae_UCG-005* at the genus level. (**K**) Box-plot of *Bilophila* at the genus level. (**L**) Box-plot of *Bacteroides* at the genus level. (**M**) Box-plot of *Parabacteroides* at the genus level. (**N**) Box-plot of *Roseburia* at the genus level. (**O**) Taxonomic cladogram derived from LEFSe analysis. (**P**) The microbiota phylotypes with statistically significant abundance differences among groups were identified by LEfSe analysis. (**Q**) The distribution proportion of predominant OTUs across various sample groups. HFD vs. Control, *p* < 0.05, #; *p* < 0.01, ##; SIM, LDOE, HDOE vs. HFD, *p* < 0.05, *; *p* < 0.01, **.

We conducted a comparison of the relative abundances of the dominant taxa at the phylum level to evaluate the particular alterations in the gut microflora. As shown in Figure 6A, the gut microbiota of each group of rats mainly consists of *Firmicutes*, *Bacteroidetes*, *Verrucomicrobia*, and *Proteobacteria*, notably with *Bacteroidetes* and *Firmicutes* constituting over 90% of the fecal microbiome composition. As depicted in Figure 6C,F, the relative abundance of *Firmicutes* and *Proteobacteria* in the guts of rats fed with LDOE and HDOE is reduced compared to the model group, while Figure 6E,G show an increased relative abundance of *Verrucomicrobia* and *Actinobacteria* in the LDOE and HDOE groups. The attributes of gut bacteria in obese humans and animals have been found to be causally related to a high *Firmicutes*/*Bacteroidetes* (F/B) ratio. As illustrated in Figure 6B, a high-fat diet substantially elevated the F/B ratio to 2.88 (*p* < 0.05), and the elevation in the F/B ratio induced by HFD was significantly diminished through treatments with SIM and HDOE (*p* < 0.05).

The box-plot in Figure 6C–G depicts the gut microbiota composition of HFD rats at the phylum level, specifically showing the presence of *Firmicutes*, *Bacteroidetes*, *Verrucomicrobia*, *Proteobacteria*, and *Actinobacteria*. In the figure, it can be seen that the HFD group has a higher relative abundance in *Firmicutes* and *Proteobacteria*, and a lower content in *Bacteroidetes* and *Verrucomicrobia*; whereas the LDOE and HDOE groups have a higher relative abundance in *Verrucomicrobia* and *Actinobacteria*.

The results in Figure 6H show that when analyzed at the genus level, the relative abundances of *Ruminococacaceae_UCG-005* and *Bilophila* in the HFD were higher than those in the control. Nevertheless, following the intervention of DOE, the relative abundances of *Akkermansia* and *Roseburia* were elevated in contrast to those in the HFD group. These results indicate that DOE intervened in the alterations of the rats’ gut microbiota caused by HFD, restoring it to a more favorable state.

The linear discriminant analysis effect size (LEfSe) method was applied to detect the species biomarkers presenting substantial abundance variations between groups. Based on the LEfSe analysis outcomes, it can be clearly seen that the principal contributing species exhibited significant differences among distinct groups, as vividly depicted in Figure 6O–Q. The gut microbial community in the HFD was mainly dominated by *Faecalitalea* and *Dorea*. In the LDOE and HDOE groups, the main contributors were *Akkermansia*, *Collinsella*, and *Rikenellaceae_RC9_gut_group*. Significantly, the HDOE group’s community was predominantly characterized by three operational taxonomic units (OTUs) of *Akkermansia*, and the proportion of *Akkermansia* in this group was significantly higher than that in other groups.

### 3.8. Effect of DOE on NF-κB/IκBα Signaling Pathway in Intestinal Tissues of HFD Rats

**Figure 7:**
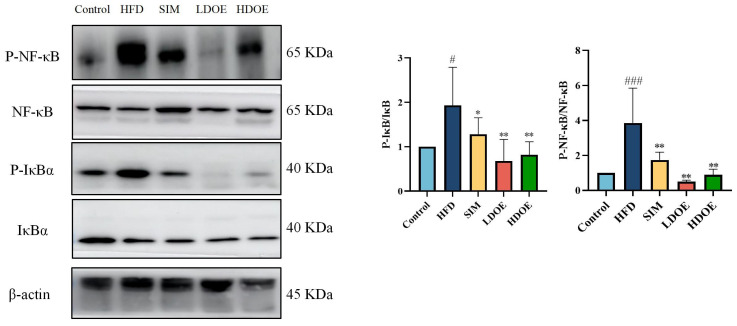
The effects of DOE on NF-κB/IκBα and their phosphorylated forms in the intestinal tissues of HFD rats. HFD vs. Control, *p* < 0.05, #; *p* < 0.001, ###; SIM, LDOE, HDOE vs. HFD, *p* < 0.05, *; *p* < 0.01, **.

To delve into the cascade response between the gut and the liver, we further probed into the expression of NF-κB and IκBα in intestinal tissues. As visually presented in Figure 7, in the intestines of rats on an HFD, the protein expression levels of NF-κB and IκBα exhibited a significant upsurge, reaching 2.30-fold and 3.86-fold, respectively, when compared with those in the control group. Respectively, treatment with DOE effectively counteracted these alterations.

In addition, when compared with rats simply fed an HFD, the protein expression levels of NF-κB and IκBα were notably elevated in the intestines of HFD-induced obese rats. Nevertheless, after DOE treatment, these levels were significantly reduced (*p* < 0.01 or *p* < 0.001).

Taken together, our findings lead us to speculate that DOE may have the potential to suppress the inflammation triggered by an HFD in intestinal tissues.

### 3.9. Correlation Analysis

**Figure 8:**
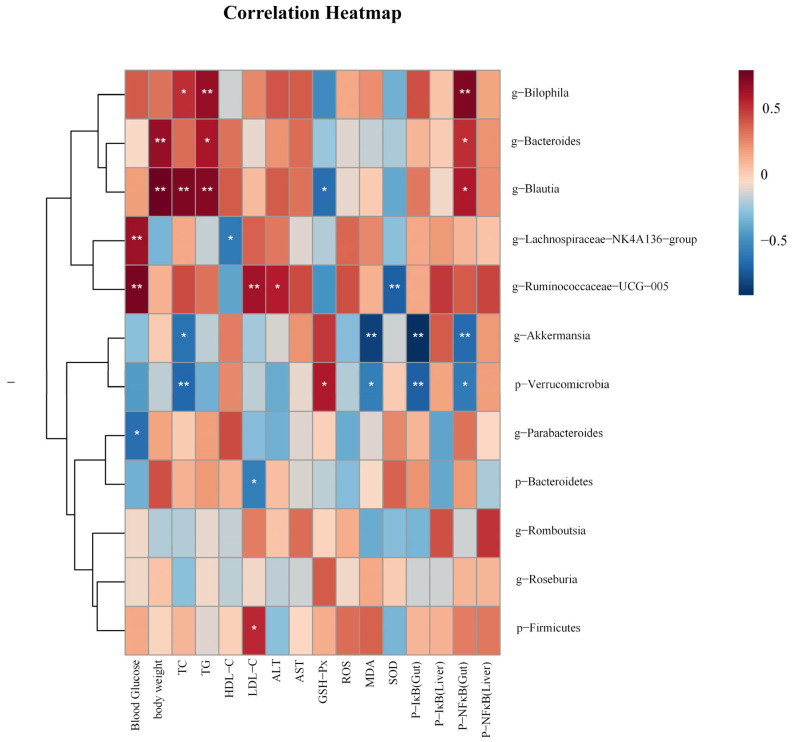
The heatmap illustrates the results of a correlation analysis between notable gut microbiota and biochemical parameters like physical measurements, oxidative stress levels, and related protein expression levels. *p* < 0.05, *; *p* < 0.01, **.

In order to investigate the correlations between gut microbiota and biochemical parameters, we employed Spearman analysis. As shown in Figure 8, *Akkermansia* was negatively correlated with TC, P-NF κB (Gut), P-IκB (Gut), and MDA, (*p* < 0.05 and *p* < 0.01). *Blautia* was positively correlated with TC, TG, Body weight, and P-NF κB(Gut) (*p* < 0.05 and *p* < 0.01), and negatively correlated with GSH-Px (*p* < 0.05). *Bilophila* was positively correlated with TC, TG, P-NF κB (Gut) (*p* < 0.05 and *p* < 0.01). Ruminococcaeae_UCG-005 was positively correlated with LDL-C, blood glucose, and ALT (*p* < 0.05 and *p* < 0.01), and negatively correlated with SOD (*p* < 0.01). Bacteroides was positively correlated with body weight, TG, and P-NF κB (Gut) (*p* < 0.05 and *p* < 0.01).

## 4. Discussion

According to recent studies, the principle of ’homology between medicine and food’, emphasizing the close ties between food and medicine, is slowly spreading across the globe [25]. Numerous foods have been found to offer both nutritional and medicinal benefits, providing not only sustenance but also supporting the body’s health and treating diseases through pharmacological means [26]. *Dendrobium officinale* is a plant with the characteristic of medicine–food homology in China. A substantial number of research works have demonstrated that the extract of *Dendrobium officinale* exhibits protective effects against oxidative stress [27], as well as liver lipid accumulation and inflammation [28]. *D. officinale* is little known regulating its anti-obesity effects and potential mechanisms of action. In this study, our objective was to uncover the possible mechanisms through which the DOE exerts its beneficial impacts. These impacts are on obesity and related metabolic syndrome. The obtained results indicated that when HFD-induced obese rats were treated with DOE, there was a notable decline in lipid droplet accumulation and a slowdown in body-weight gain. Moreover, DOE treatment played a significant role in alleviating the systemic inflammatory response in these obese rats. The hepatic oxidative stress induced by the HFD was also markedly suppressed in obese rats after they were administered with DOE. Our findings further suggested that the consumption of DOE led to an elevation in the relative abundances of probiotic bacteria in HFD-induced obese rats. Furthermore, DOE exerts anti-inflammatory effects by suppressing the NF-κB/IκB signaling pathway, which plays a crucial role in ameliorating the overall metabolic profile of rats.

A sustained high-fat diet has the potential to precipitate metabolic disruptions. These disruptions are likely to trigger visceral obesity and the abnormal deposition of triglycerides in ectopic sites, such as hepatic steatosis, and simultaneously augment oxidative stress within the liver [5]. Numerous investigations have documented that an HFD has the capacity to trigger aberrant lipid accretion. This abnormal lipid accumulation subsequently results in the progression of obesity and hyperlipidemia [29]. For instance, the liver, a key organ in lipid metabolism, is over-burdened with processing the excess fat. This can lead to non-alcoholic fatty liver disease (NAFLD), where fat droplets accumulate in liver cells, impairing liver function and potentially progressing to more severe liver conditions such as cirrhosis. Moreover, the chronic low-grade inflammation induced by abnormal lipid accumulation is involved in the initiation and progression of insulin resistance [30]. Insulin resistance serves as a forerunner to type 2 diabetes. This is because the body’s cells become less sensitive to the effects of insulin, which in turn results in increased blood sugar levels. Compared with HFD rats, those receiving DOE exhibited a decrease in serum TC and TG levels, along with an increase in HDL-C levels. Histological observations indicated that after DOE treatment, the hepatic vacuolization state and the number of lipid droplets had been similar to those of the control group. Oxidative stress represents a condition where the equilibrium between the oxidative and antioxidant systems within cells and tissues is perturbed [31]. As a result, the level of ROS is elevated, and lipids, proteins, and DNA are damaged by these ROS [32,33,34]. The oxidative breakdown process of polyunsaturated fatty acids within cells leads to the formation of malondialdehyde (MDA) as one of its end products. When there is an upsurge in free radicals, it leads to the excessive generation of MDA [35]. SOD [36] and GSH-Px exert significant functions in counteracting oxidative stress. Oxidative stress in the liver holds a pivotal position in the pathogenesis of hyperlipidemia [37]. In our study, intake of DOE diminished the levels of hepatic MDA and ROS, and augmented the levels of antioxidant enzymes (GSH-Px and SOD) to counteract the damage induced by HFD, which is analogous to the results of previously reported studies that utilized Dendrobium officinale in their treatments [38,39].

Chronic diseases such as obesity can give rise to hyperlipidemia, cause abnormal blood lipid levels, and thus trigger inflammation [40,41]. The transcription factor NF-κB can exert regulatory effects on the functions of innate and adaptive immunity, and acts as a key mediator of the inflammatory response [42]. Upon exposure of cells to inflammation-associated stimuli, like bacterial lipopolysaccharide (LPS) and pro-inflammatory cytokines (for example, tumor necrosis factor-α (TNF-α) and interleukin-1 (IL-1)), the intracellular signal transduction pathways become activated. When the IκB kinase (IKK) acts on IκB, it induces phosphorylation of IκB. This phosphorylation event then triggers the ubiquitination and degradation of IκB. As a result, NF-κB is released and translocates into the nucleus, where it binds to specific DNA sequences and initiates the transcription of related genes, including those encoding pro-inflammatory cytokines (e.g., TNF-α, IL-1, IL-6) and chemokines (e.g., IL-8). The massive production of these inflammatory mediators further amplifies the inflammatory response and exacerbates tissue damage. When NF-κB is dysregulated in intestinal epithelial cells and immune cells, its abnormal operation can undermine the integrity of the intestinal mucosal barrier and set off long-term intestinal inflammation. Activating the dysregulated NF-κB helps to modulate the pathogenic processes of multiple inflammatory diseases [32]. In our study, the DOE can reduce inflammation in the liver and intestinal tissues of HFD rats by regulating phosphorylated NF-κB and phosphorylated IκB, thus achieving an anti-inflammatory effect. Consistent with the previously reported results, the extract of Dendrobium officinale can exert its anti-inflammatory activity by inhibiting the NF-κB signaling pathway [23].

Among obese humans and animals, it can be observed that the gut bacterial makeup is related to a significantly elevated *Firmicutes*/*Bacteroidetes* (F/B) ratio and a notably diminished diversity of the gut microbial community [43,44]. In this study, we discovered that the treatment with HDOE significantly lowered the F/B ratio and ameliorated the reduction of microbial species richness in HFD-induced obese rats. Additionally, the treatment with DOE led to a decrease in the abundance of *Bilophila*, while the abundances of *Akkermansia* and *Roseburia* were increased. Previous reports have indicated that *Akkermansia* can reverse the adverse symptoms induced by an HFD in obese rats. These adverse symptoms include an increase in fat mass, endotoxemia, and inflammation [45]. Additionally, it was discovered that there was a substantial interconnection between *Akkermansia mucinphila* and the expression levels of markers relevant to lipid metabolism processes (fatty acid oxidation markers: *Acox1, Cpt1a, Acacb*) and inflammation (metabolic inflammation markers: *Tnf*, *Ccl2*, *Itgax*, *Emr1*, *Lbp*, *Il6*, *Il1*)-mediated reactions within adipose tissue. Additionally, it was also closely associated with several circulating parameters (i.e., glucose, insulin, triglycerides, leptin) in diet-induced obesity (DIO) mice [46]. Extensive effects of *Roseburia* and its derivatives have also been found to play a role in immune system modulation and inflammatory response regulation [47]. The bacterium *Roseburia*, which is anaerobic, Gram-positive, and slightly curved with a rod shape and flagella, creates butyrate in the colon. Increasing evidence has strongly suggested that *Roseburia*, a prominent gut microorganism, can effectively combat intestinal inflammation and preserve energy stability. This is accomplished by the synthesis of particular metabolites that are essential for adjusting the intestinal milieu and metabolic activities. *Roseburia* primarily functions as a primary butyrate-producing bacterium [47]. Butyrate produced by commensal microbes was revealed to promote the proliferation of extrathymic regulatory T cells (Tregs) through intrinsic enhancer conserved non-coding sequence 1 (CNS1). Tregs, as vital anti-inflammatory lymphocytes, produce interleukin-10 (IL-10), transforming growth factor beta (TGF-β), and interferon gamma (IFNγ). Research has shown that Roseburia inhibits the secretion of interleukin-17 (IL-17) and promotes the differentiation of Tregs in colitis induced by 2,4,6-trinitrobenzenesulfonic acid (TNBS) [48]. Nevertheless, the reduction in *Bilophila* following DOE treatment might exert a beneficial impact on obesity. This is due to the fact that elevated levels of *Bilophila* species are capable of exacerbating inflammation and metabolic dysfunctions in HFD-induced obese rats; these bacteria may disrupt the normal gut microbiota balance, leading to increased endotoxin production. The endotoxins can enter the bloodstream, triggering a systemic inflammatory response. Moreover, they may interfere with insulin signaling pathways, further worsening insulin resistance and lipid metabolism disorders in these obese rats [49]. This indication shows that the gut microbiota may serve as a key area for the positive health outcomes brought about by DOE.

In our study, when considering the genus level, in contrast to the control group, the abundances of *Akkermansia* and *Roseburia* were elevated in both the LDOE group and HDOE group. Conversely, the abundance of *Ruminococcaceae_UCG-005* was reduced. Correlation analysis showed that the abundance of *Akkermansia* was negatively correlated with diseases, which is consistent with previous reports. As a novel biotherapeutic agent, *Akkermansia* can induce the expression of mucins MUC2 and MUC3 [50], thereby preventing the adhesion of enteropathogenic Escherichia coli (EPEC) to the epithelium and improving health by regulating various metabolic disorders [51]. Our study shows that by supplementing with DOE, the abundances of *Akkermansia* and *Roseburia* in the gut can be increased, and targeting *Akkermansia* can alleviate the glycolipid metabolic disorders and inflammatory effects induced by an HFD. Our findings furnish a scientific rationale for treating DOE as a prebiotic, highlighting its potential to modulate gut microbiota and support overall health through prebiotic-like mechanisms.

## 5. Conclusions

**Figure 9:**
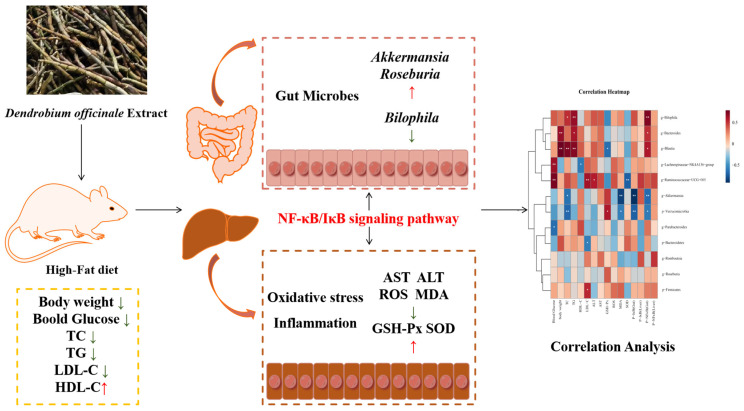
DOE alleviates liver and intestinal inflammation in rats induced by high-fat factors by regulating the gut microbiota. *p* < 0.05, *; *p* < 0.01, **.

To sum up, as vividly shown in Figure 9, the findings of this study highlight that DOE has a remarkable ability to lower the blood glucose and lipid levels in HFD rats. Additionally, it can enhance the alleviation of oxidative stress-induced injury in the liver. Additionally, DOE can regulate the composition of the gut microbiota, inhibit the proliferation of harmful bacteria, and promote the proliferation of probiotics. Moreover, the supplementation of DOE has a notably suppressive effect on the NF-κB and IκB signaling pathways, thereby suppressing inflammation in the liver and intestine. This provides a new approach for the precise dietary intervention of *Dendrobium officinale* in improving metabolic syndrome.

## Data Availability

Data are contained within the article.

## Supplementary Materials

The following supporting information can be downloaded at https://www.mdpi.com/article/10.3390/antiox14040432/s1.
