# Supplementary material for: Anti-Inflammatory Effect of Dendrobium officinale Extract on High-Fat Diet-Induced Obesity in Rats: Involvement of Gut Microbiota, Liver Transcriptomics, and NF-κB/IκB Pathway
Source: Antioxidants (Basel). 2025 Apr 3;14(4):432. doi: 10.3390/antiox14040432 (PMC12024317; doi:10.3390/antiox14040432)
Supplement: Supplementary file 1 [file antioxidants-14-00432-s001.zip › antioxidants-3539965-supplementary.pdf]

(A)

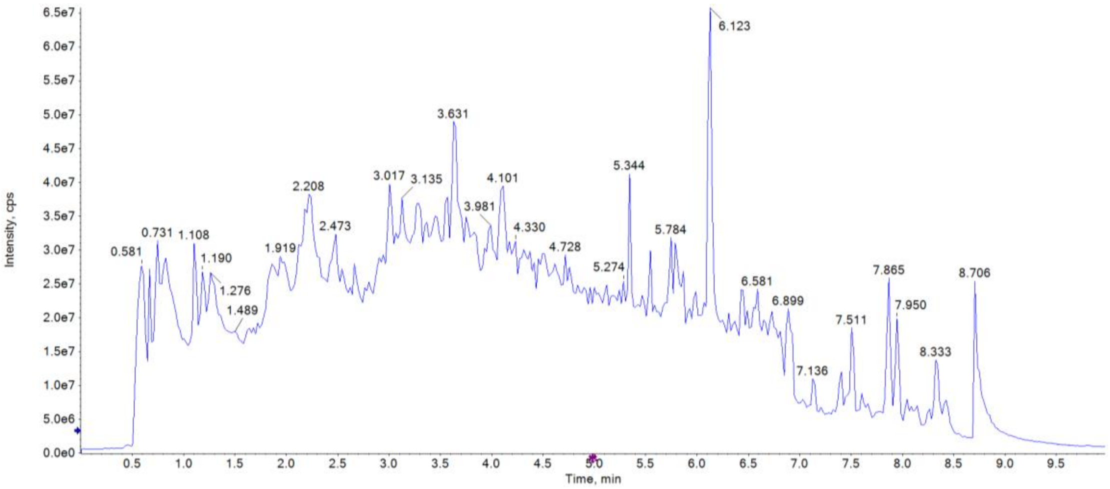

(B)

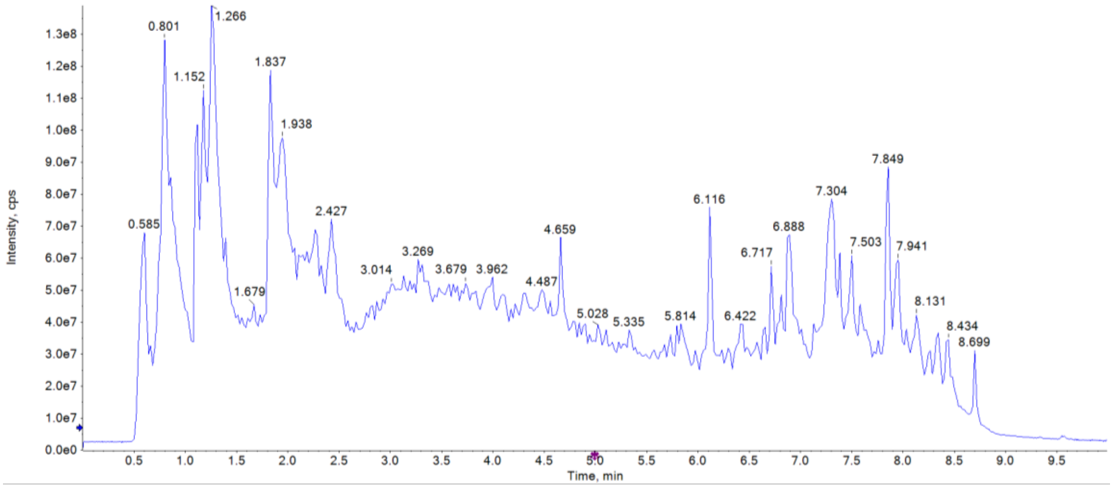

Figure S1: The total ion chromatogram (TIC) of DOE. (A) The negative ion chromatogram of DOE, (B) The positive ion chromatogram of DOE.

Table S1. The composition of the cold extraction solution in each reagent kit

Name	Catalog numbers	Extract Components
ALT	GPT-1-Y	$Na_2HPO_4\cdot 12H_2O$ and $NaH_2PO_4\cdot 2H_2O$ buffer
AST	GOT-1-Y	$Na_2HPO_4\cdot 12H2O$ and $NaH_2PO_4\cdot 2H_2O$ buffer
CAT	CAT-1-W	$Na_2HPO_4\cdot 12H_2O$ and $NaH_2PO_4\cdot 2H_2O$ buffer

GSH-Px	GPX-1-Y	$Na_2HPO_4 \cdot 12H_2O$ and $NaH_2PO_4 \cdot 2H_2O$ buffer
SOD	SOD-1-W	$Na_2HPO_4 \cdot 12H_2O$ and $NaH_2PO_4 \cdot 2H_2O$ buffer
ROS	ROS-1-Y	Tris - HCl buffer with sucrose and EDTA<br>Tris - HCl buffer with KCl, $MgCl_2 \cdot 6H_2O$, and glucose<br>Phenylmethylsulfonyl fluoride (PMSF) solution

Table S2. 10 compounds in DOE including Flavonoids, Terpenoids, Alkaloids.

S.NO	Compound	RT (Retention Time) (min)	Molecular formula	Molecular weight	Class of compound
1	Quercetin	0.5924	C15H10O7	302.0427	Flavonoids
2	Maclurin	0.8826	C13H10O6	262.0477	Flavonoids
3	Silicristin	2.1457	C25H22O10	482.1213	Flavonoids
4	6-Hydroxyflavone	2.2648	C15H10O3	238.063	Flavonoids
5	Pelargonidin 3-O-beta-D-sambubioside	3.7296	C26H29O14+	565.1557	Flavonoids
6	Neoschaftoside	3.7312	C26H28O14	564.1479	Flavonoids
7	Rutin	4.1923	C27H30O16	610.1534	Flavonoids
8	Vitexin-2''-O-glucoside	4.1923	C27H30O16	610.1534	Flavonoids
9	Lonchocarpol A	4.4136	C25H28O5	408.1937	Flavonoids
10	Apigenin	5.5459	C15H10O5	270.0528	Flavonoids
11	Specioside	0.9219	C24H28O12	508.1581	Terpenoids
12	Amarogentin	1.1784	C29H30O13	586.1686	Terpenoids
13	Lettucenin A	1.1855	C15H12O3	240.0786	Terpenoids
14	Vernoflexin	1.6075	C20H24O4	328.1675	Terpenoids
15	Polyporusterone A	1.6512	C28H46O6	478.3294	Terpenoids
16	Reciniferatoxin	1.7471	C37H40O9	628.2672	Terpenoids
17	Acetylvalerenolic acid	1.7671	C17H24O4	292.1675	Terpenoids
18	Bruceantarin	1.8304	C28H30O11	542.1788	Terpenoids
19	Vernoflexin	1.6075	C20H24O4	328.1675	Terpenoids
20	Pseudaconine	2.245	C25H41NO8	483.2832	Terpenoids

21	Specioside	0.9219	C24H28O12	508.1581	Terpenoids
22	Aknadicine	1.125	C19H23NO5	345.1576	Alkaloids
23	Nb-Feruloyltryptamine	1.1257	C20H20N2O3	336.1474	Alkaloids
24	Daurioxoisoporphine B	1.1257	C19H16N2O4	336.111	Alkaloids
25	Latifoline	1.1291	C20H27NO7	393.1788	Alkaloids
26	Dopamine	1.139	C8H11NO2	153.079	Alkaloids
27	Symlandine	1.139	C20H31NO6	381.2151	Alkaloids
28	N-Acetylputrescine	1.1489	C6H14N2O	130.1106	Alkaloids
29	Arecoline	1.671	C8H13NO2	155.0946	Alkaloids
30	Aristolodione	1.671	C18H13NO4	307.0845	Alkaloids
